# Rasch Analysis of the Profiles of Occupational Engagement in people with Severe mental illness (POES) instrument

**DOI:** 10.1186/s12955-015-0327-0

**Published:** 2015-08-19

**Authors:** Ulrika Bejerholm, Åsa Lundgren-Nilsson

**Affiliations:** Medical Faculty, Department of Health Sciences, Work and Mental Health, Lund University, Lund, Sweden; Department of Clinical Neuroscience and Rehabilitation, the Sahlgrenska Academy, University of Gothenburg, Gothenburg, Sweden

## Abstract

**Purpose:**

The Profiles of Occupational Engagement in people with Severe mental illness (POES) instrument was developed to study time use profiles of occupations and measure the extent they are characterized by engagement. However, the dimensional factors are not known. The aim of the present study was to establish the internal construct validity of the POES using the Rasch measurement model.

**Methods:**

A sample of 192 outpatients in Sweden was administered the POES and data were subjected to Rasch analysis.

**Results:**

The POES showed good fit to the Rasch model after accommodation for local dependency. The nine items had high reliability as measured by person separation index, and no threshold disordering was present. Differential item functioning analysis showed no significant differences across groups of age, sex, diagnosis, or country of origin.

**Conclusion:**

The POES is a unidimensional scale that represents a continuum of occupational engagement. The transformed POES sum score can be used on an interval scale to measure status and changes in occupational engagement in mental health practice and research.

## Background

Having a severe mental illness affects one’s entire existence during all hours of the week. To understand what people with severe mental illness do with their time, what engages them, what they perceive as meaningful or not, how they interact with others and navigate in society is important in order to understand their health, quality of life and well-being, and to support their occupational goals [1]. Occupation refers to our doing in time and place, all activities we occupy ourselves with and not only work [2]. The connection between time use and health is fundamental [3, 4]. Time use methodology, such as time use diaries, is one of the most prominent methods to understand humans as occupational beings [4, 5]. However, no specific guidelines exist for how to interpret the information produced by a time use diary, and the information obtained is rich, unstructured, and not easily concluded or communicated in a consistent way [6, 7]. Consequently, the time use diary cannot be used as an independent outcome measure for the target group. For this reason, the Profiles of Occupational Engagement in people with Severe mental illness (POES) was developed [6]. The time use diary constitutes the basis for the assessment of occupational engagement in POES and provides information on the occupational, environmental, and occupational domains of client occupational performance. The occupational engagement construct includes both objective and subjective aspects of occupational performance. Qualitative time use research, through which the POES was developed, shows that people with severe mental illness engage in occupations to varying extents [8, 9]. Occupational engagement is expected to increase and decrease along a continuum. This understanding formed the basis for the POES assessment categories. Time use research further helped to identify nine occupational engagement themes or items categories that varied in a systematic way in relation to the level of occupational engagement. Together they constitute elements of the construct [6]. As a whole, the POES helps to identify imbalances between activity and rest, lack of meaningful occupations and few occupational and environmental opportunities, and set relevant occupational goals. In this sense, POES can help to plan and evaluate occupational interventions in mental health practice.

Subsequently, the POES was used in research. The critical relationship between occupational engagement and health related factors can now be studied. Cross-sectional studies show that a higher level of occupational engagement is related to better quality of life, mastery, internal locus of control, fewer symptoms [1], occupational balance [10], increased work motivation [11], and empowerment [12]. In a randomized controlled trial of 120 participants with severe mental illness of the effectiveness of the Individual Placement and Support approach to vocational rehabilitation, POES was sensitive to changes between measurement points (0, 6 and 18 months) and intervention groups [13]. However, in a pilot randomized controlled 12-week trial with 24 participants of the effectiveness of an intervention entitled “Action Over Inertia”, no changes in occupational engagement were detected [14]. Traditional psychometric testing of POES supports inter-rater reliability, internal consistency [6], and external construct validity [15]. However, the POES scaling is ordinal, parametric statistics cannot be done, and the raw score cannot be transformed into an interval scale. This is a limitation since health outcomes are frequently used for evaluation of clinical trials where valid change scores and access to parametric statistics are required [16].

Mathematical psychometric approaches supplement traditional psychometric testing [17]. The Rasch measurement model is such an approach used to investigate the psychometric properties of outcome scales, and considered to capture a unidimensional construct [18]. The Rasch measurement model helps to disclose a lack of variance that is not easily detected with traditional psychometric testing [19]. Over the past 20 years, Rasch analysis has been increasingly used in health sciences for development and evaluation of questionnaires, scales and measures [20, 21]. Rasch analysis can be used when a new scale is being developed, when reviewing the psychometric properties of existing scales [22]. A unique feature of the model is that if the scale fits the model, no assumptions about the distribution of participants on the scale needs to be made during statistic calculations [23]. In the present study, the Rasch model is used to determine the POES item fit to the model, reliability, threshold ordering, differential item functioning (DIF), local response dependency, and unidimensionality. The underlying construct of occupational engagement is assumed to reflect lower and higher levels of client occupational engagement. The test of fit to the Rasch model indicates whether the hierarchical and appropriate ordering of the items, i.e., probabilistic ordering [24], varies across the trait of occupational engagement, comprising required properties of variance. For each POES scale item, clients with little engagement in occupations would be rated lower; clients with more engaged time use would be rated higher. The reliability test evaluates if all POES items are complementary and related by drawing a more complete picture of the unidimensional construct of occupational engagement. The threshold ordering analysis involves each category response for each POES item and shows whether or not an ordered set of response thresholds (distance across items across the trait) is present. Differential Item Functioning (DIF) can be thought of as item bias, and the test helps to study whether different groups (i.e., age, sex, and cultural groups) respond in different manners [22]. This means that the expected score on any of the POES items should be the same, irrespective of group identity. In addition to the groups mentioned, diagnosis groups are included in the present DIF analysis as is consistent with previous research [25]. People with psychosis may be assumed to have certain problems in asserting their lives compared to those with a less fragmented self-perception [26]. In local response dependency, items are linked so that response to one item will depend upon the response to another item. The presence of response dependency inflates reliability, compromises parameter estimation, and can be detected through the correlation of residuals. With regard to the POES, items are expected to be closely connected since the items collectively constitute parts of the occupational performance of a client in real life situations. However, each item area has high clinical relevance. Each item informs the therapist and client about whether an engagement area needs further attention to understand the composition of a client’s disability and occupational goals. For example, an item that concerns the extent to which a client can be present in social environments is related to the ability for social interplay, i.e., being socially responsive and collaborating with others in a reciprocal relatedness. And, each area may need to be targeted differently for clinical purposes [15]. Lastly, the unidimensionality of POES needs to be tested to ensure that the sum of items forms a unidimensional scale, as this is a basic prerequisite for combining any set of items into a total score. A more detailed description of Rasch analysis process can be found in studies by Tennant and Conaghan [22] and Hagquist, Bruce and Gustavsson [19]. The POES is used worldwide and demonstrates satisfactory psychometric properties through traditional measurements. However, the dimensional factors are not known and the raw score cannot be transformed into interval scaling to be used in parametric statistics to evaluate change scores. The aim of the present study was to establish the internal construct validity of the POES, using the Rasch measurement model.

## Methods

### Research design

This psychometric study evaluates the POES internal construct validity using the Rasch measurement model among people with severe mental illness who live in a southern Swedish city. Two data sets were used [10, 27]. We obtained approval from the Regional Ethical Committee in Lund, and each participant completed written consent. Study procedures were in accordance with the Helsinki Declaration.

### Participants and procedures

People with severe mental illness were recruited from three outpatient units for this target group in a city with a population of 300 000. In this city, more than one in four citizens originates from another country. The units specialize in meeting the needs of people with severe mental illnesses and disabilities. In this study, severe mental illness refers to having a psychosis or severe psychiatric disability that extends over at least two years [26]. Diagnoses were validated against the medical record, and illness or disability severity was assessed by the team psychiatrist. Other inclusion criteria were receiving mental health services, residence in the community, aged 18–63 years, and the ability to understand, speak, and write Swedish. With support from the outpatient unit staff, written and oral information was provided to potential participants. Each person gave their written consent to research assistants, who later set up interview appointments and collected the data. The research assistants were registered occupational therapists.

### Data collection

A questionnaire on sociodemographic and clinical characteristics was used to collect background information, i.e., age, age at first contact with psychiatry, sex, civil status, country of origin group, and diagnosis. Psychiatric symptoms were assessed with the Brief Psychiatric Rating Scale (BPRS) by trained assessors who work at the outpatient units. BPRS consists of 18 items that are rated on a seven-point scale based on an interview and behavior as observed during the last two weeks. A high score indicates more symptoms. The items include disorganization, disorientation, depressive symptoms, and hostility. They allow for analysis of positive, negative, and depressive symptoms, and general psychopathology. Good inter-observer and intra-observer reliability have been demonstrated [28, 29]. Cronbach’s alpha coefficient was .78.

### The Profile of Occupational Engagement in persons with Severe mental illness (POES)

The POES was developed with qualitative research based on time use in relation to the constructs of occupational performance [8] and occupational engagement [6, 9]. The pilot version was systematically refined by occupational therapy clinicians and researchers from Sweden and the UK [6]. The POES consists of two parts, data collection and assessment scale, and is administrated by an occupational therapist. In this sense, the POES has the same architecture as the Worker Role Interview (WRI) or the Work Environment Impact Scale (WEIS) [30]. In WRI and WEIS, data collection (structured interview) precedes the assessment. The Rasch analysis involves the assessment part, or POES scale. Data collection involves the client filling in a 24-h yesterday time use diary sheet, divided into four columns with one-hour intervals. Each column has a question at the top, the first asks about occupations performed, the second and third questions are about the social and geographical environments, and the fourth is about personal reflections, emotions, and comments on the performance. In this way, a horizontal segment in the diary represents dimensions of the occupational, environmental, and personal domains of occupational performance [31]. Thus, a completed diary sheet represents several modules of occupational performance that make up a day [8]. If necessary, a supplementary interview is performed. This is based on dialogue with the client about what is written in (or left out of) the diary. The interview serves as a memory aid and helps the client recall past events. The assessment of occupational engagement is based on the completed time use diary and is performed by an occupational therapist with previous training in POES use. The nine items are: Daily rhythm of activity and rest, Place, Variety and range of occupations, Social environment, Social interplay, Interpretation, Extent of meaningful occupations, Routines, and Initiating performance, which are rated on a four-point scale [6]. A detailed text supplements each item score to help the assessor differentiate between the ranking categories. A higher score indicate a higher level of occupational engagement. Traditional psychometric testing has shown satisfactory content validity, good internal consistency (Cronbach’s alpha = .95), and inter-rater agreement (overall mean weighted kappa = .78) [6]. External construct validity in relation to global psychosocial functioning (GAF, *rs* = .73) and self-assessed activity level, and satisfaction with daily occupations in people with severe mental illness (*rs* = .70) have also been shown [15].

### Rasch analysis

The Rasch measurement model, Partial Credit version [32], was used to analyze the psychometric properties of the POES using RUMM 2030 software [33]. To test the overall fit of the POES items to the Rasch model, an overall summary statistics were evaluated with *Χ*^2^ statistics with non-significant *Χ*^2^ probability values after Bonferroni adjustment, and a mean person and item fit residuals score of 0 ± 1.0 (SD) indicate a good model fit. A significant *Χ*^2^ reflects that the hierarchical ordering of the POES items varies across the measured trait (occupational engagement). The fit of individuals and items were assessed with non-significant *Χ*^2^ probability values; standardized fit residuals of −2.5 to +2.5 indicate adequate fit (CI, 99 %) [23]. Reliability was tested by use of a person separation index (PSI). PSI indicates how many distinct groups of people (statistically defined) the category scale of POES is able to separate between, and thus the power of the construct to discriminate among respondents. PSI is analogous to Cronbach’s alpha when data are normally distributed. Both PSI and Cronbach’s alpha (without missing data) identify the measure of reliability with .70 being the lowest level of acceptability (group use) [22, 34]. Appropriateness of the response categories (threshold ordering) was also examined [22]. This ensures that the increase of a POES item response category, as represented by the thresholds between ranking categories, reflects an increase in the level of occupational engagement (i.e., 1-2-3-4). Disorder thresholds may be related to ambivalent wording or too many categories [33]. Differential Item Functioning (DIF) is formal test of invariance that shows whether group membership with similar levels of the investigated trait (occupational engagement) respond to the items in a systematic way [35]. The presence of DIF (bias for an item among sample subgroups) was evaluated for sex (men/women), age group (20 to 39 years/40 to 58 years), diagnostic group (psychosis/other), and country of origin (Sweden/other countries). Local response dependency [36] between items was assessed with the residual correlation matrix for pairs of items whose correlation values were greater than .30 [23]. When high residual correlations were detected (i.e., dependency between items), the items were combined or summed into a “testlet” (super item) [37, 38]. This made it possible to account for the possible violation of local dependency, data were re-analyzed using the combined correlated items (testlet) and comparing the fit with that provided by the initial analysis. A reliability estimate was obtained that compared the testlet with the original value [38]. The local response dependency can thus be accommodated by the calculation of testlets and does not impair the ability of a scale to measure a common dimension [21]. Unidimensionality of the POES instrument was assessed by performing a principal component analysis of the residuals to identify subtests of items that might reflect a further dimension. Person estimates from these subtests were compared using a series of independent *t*-tests. If less than 5 % of the tests are significant, the scale is considered unidimensional [22, 39]. A 95 % binomial confidence interval of proportions can be used to show that the lower limit of the observed proportion is below the 5 % level [39].

## Results

### Participants

A total of 192 participants were included. As shown in Table 1, most were single, and slightly more participants were men. About 30 % originated from another country. The average age was 39 years. Although 78 % had psychosis and contact with the outpatient unit for a longer period of time, according to the BPRS they did not exhibit symptoms associated with an acute or active phase of illness. People with schizophrenia or other psychotic disorders were included in the “psychosis” diagnostic group. Bipolar disorder, depression, social phobia, and personality disorder were represented among participants in the group "other". There was an even distribution of participants among the disorders.

**Table 1:** Sociodemographic data of study participants

		*n* = 192
Age, mean (*SD*), min-max		39.4 (8.5), 21-58
Age first contact psychiatry, mean (*SD*), min-max		25.5 (8.5), 12-56
Sex		
	Males, *n* (%)	116 (60.4)
	Females, *n* (%)	76 (39.6)
Civil status		
	Single, *n* (%)	124 (64.9)
	Divorced, *n* (%)	27 (14.1)
	Married or partnership, *n* (%)	26 (13.6)
	Other, *n* (%)	7 (3.7)
Ethnicity		
	Native (Swedish), *n* (%)	135 (70.3)
	Europe, *n* (%)	26 (13.5)
	Africa/Middle East/Asia, *n* (%)	31 (16)
Psychopathology (BPRS; range 1–7) mean (SD), min-max		1.8 (.5), 1-3.3
Diagnosis group (*n* = 191)		
	Psychosis (ICD-10: F20-29), *n* (%)	149 (77.6)
	Other (ICD-10: F30-31, F32, F40, F60), *n* (%)	42 (21.9)

*SD* standard deviation, *BPRS* Brief Psychiatric and Rating Scale, *ICD-10* International Statistical Classification of Diseases and Related Health Problems - Tenth Revision, *F20-29* Psychotic disorders, *F30-31* Bipolar disorder, *F32* Depression, *F40* Phobia disorder, *F60* Personality disorder

### Rasch analysis

**Fig. 1:**
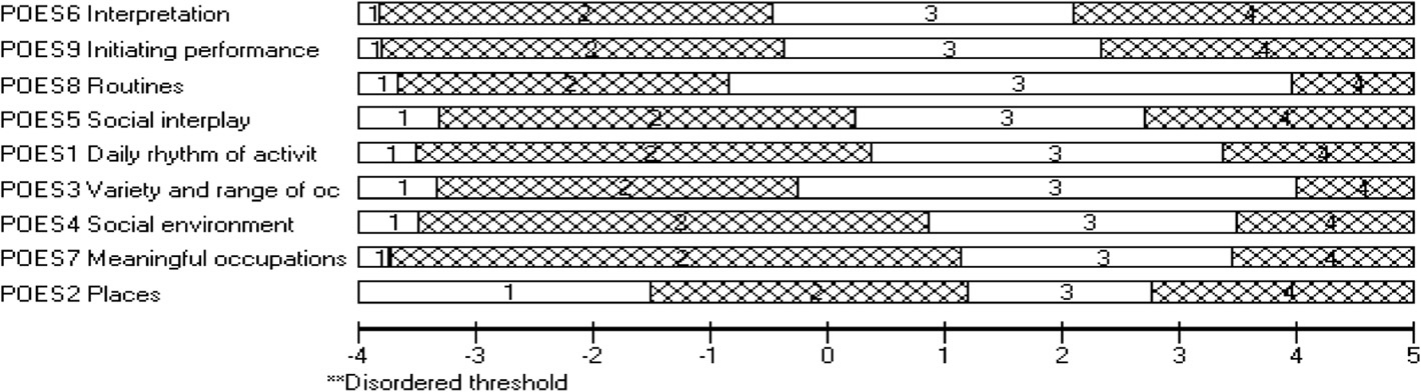
The item threshold for the 9 Profiles of Occupational Engagement in people with Severe mental illness (POES) items in order of location, from most difficult to least difficult item

The results on the POES fit to the Rasch model is presented in Table 2. The initial analysis (POES 1) showed overall fit to the Rasch model (*χ*^2^ = 18.815; *df* = 18; *p* = .40). Reliability was also high (person separation index (PSI) with extremes = .93; Cronbach’s alpha = .95). The overall person-fit (mean = −.306, *SD* = 1.22) was also acceptable, but there were indications of some misfit of items (mean = −.081, *SD* = 1.553). No threshold disordering (Fig. 1) or individual misfit of individual POES items were found, with item fit residuals within the recommended confidence range of ±2.5. With regard to item hierarchy (Fig. 1), item 6 (Interpretation) and then item 9 (initiating performance) were the most difficult items. Item 2 (Place) was the least difficult item.

**Table 2:** Fit of the Profiles of Occupational Engagement in people with Severe mental illness (POES) to the Rasch model

		Item residual		Person residual		Chi square		PSI	Unidimensional test % (CI)
Analysis name	# of items	Mean	±SD	Mean	±SD	Value	*p*		
POES 1	9	−.08	1.55	−.31	1.22	18.82	.403	0.93/0.95	8.77 (5.5–12)
POES 2	Testlet	.02	1.43	−.28	1.11	13.4	.495	0.93/0.93	7.6 (4.3–10.9)
Ideal Values		0.0	<1.4^a^	0.0	<1.4^a^		>.05^b^	>0.85	LCI <5 %

*CI* Confidence interval, *LCI* Lower CI

^a^May be higher when unequal length testlets present

^b^Boferroni-adjusted

**Fig. 2:**
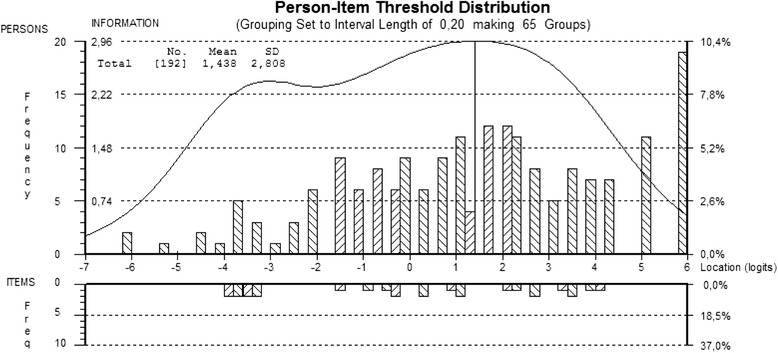
The person-item threshold distribution for the 9 Profiles of Occupational Engagement in people with Severe mental illness (POES) items

The person-item threshold distribution targets locations of persons (top histogram) relative to response category thresholds (bottom histogram) on a common logic metric (Fig. 2) indicates that the sample was located at a higher level of occupational engagement than the average of the 9 items (4 response options) (*n* = 192; mean = 1.438, *SD* = 2.808).

There was no significantly different item functioning (DIF) (*p* = .005 Bonferroni-adjusted) for the investigated groups (sex, age, diagnosis, country of origin) in any of the POES items. The principal component analysis of residuals indicated problems with the 95 % binominal confidence interval of proportions above the 5 % level in the scale (Table 2). The residual correlation matrix gave an indication of response dependency between three items (4: Social interaction, 5: Social interplay, 6: Interpretation).

Given the indicated local dependency, dependent items 4, 5, and 6 were made into a testlet (Table 2, POES 2 analysis). This resulted in a solution with still an acceptable overall fit (*χ*^2^ = 13.40; *df* = 14; *p* = .5495), good reliability by PSI with extremes = .93 and Cronbach’s alpha = .93. There were no individual item misfits with item fit residuals within the range of ±2.5. All thresholds were also still ordered, indicating that the categories work as intended. No significant DIF (*p* = .007, Bonferroni-adjusted) for any of the groups under investigation for this solution. Further tests of unidimensionality of the scale supported the unidimensionality of POES when retaining all items, since the CI lower limit was below the 5 % level. Given the fit to the Rasch model, a raw score-interval scale conversion table was created (Table 3). The scale can be used (when all items are answered) by adding up the item responses and comparing it to the metric equivalent of the raw score.

**Table 3:** Transformation of raw scores to metric scores for Profiles of Occupational Engagement in people with Severe mental illness (POES)

Raw score	Metric score
9	9
10	11
11	12
12	13
13	14
14	15
15	16
16	17
17	18
18	19
19	20
20	20
21	21
22	22
23	23
24	24
25	25
26	26
27	27
28	27
29	28
30	29
31	30
32	30
33	31
34	32
35	34
36	36

## Discussion

Based on the Rasch analysis, the POES exhibited internal construct validity. These findings uphold previous traditional psychometric testing [6, 15]. The sum score can be used with the POES scores transformed into an interval scale that represents a logical continuum in relation to the underlying construct of occupational engagement.

The local response dependency was accommodated by combining items 4, 5, and 6 into a testlet. This solution is useful from a measurement point of view, and satisfies the underlying theory of the POES scale as it keeps all of the items. The testlet can be further clarified in relation to the underlying assumption or theory of occupational engagement that forms the basis of the POES. The items 4 and 5 can be anticipated to represent the quantitative (item 4) and qualitative (item 5) aspects of social engagement and social functioning. Accordingly, these items fit together logically. The better a person is in communication and interaction skills (item 5), the more the person seeks out social environments (item 4). In terms of the interconnection between the socially-related items and item 6 (Interpretation), which represents a cognitive construct, item 6 is assumed to be closely linked to the social aspect of performance. The ability to cognitively store information and interpret what is happening socially in occupations is important to the ability to interact socially [40]. People with schizophrenia commonly misinterpret social cues and have difficulty communicating effectively with others [41]. The cognitive function of recognizing, understanding, and processing social interactions is referred to as social cognition [40]. Therefore, the construct of social cognition may help explain the dependency among the three items. Together, these items underscore the occupational engagement construct, especially since social skills and processing of social environment are significantly related to daily functioning [42] and role functioning in the community [40]. Furthermore, two recently submitted studies on cognitive functioning in relation to social skills and occupational engagement, by Lexén and Bejerholm, support the aforementioned assumption of a relationship between items. The question remains as to why the three items are not collapsed into one. As stated in the introduction, the relevance of keeping the items separate is important for clinical utility. Each item informs the occupational therapist and client on problems related to the ability to engage in daily occupations, needed goals, and how interventions should be planned. To sum up, the testlet solution resulted in maintaining the integrity of the scale for use in rehabilitation (clinimetric), while satisfying the standards of modern measurement at the same time [21, 43].

The four-point rating scale functions well and was used as intended. A similar finding was seen in a previous study in which a linear trend or *a priori* ordering (ascending or descending) was hypothesized and found in relation to different levels of occupational engagement by the Jonckheere-Terpstra test [1]. Furthermore, the initial content validation process [6] found that the POES is relevant and has coverage as the items represent a sample of behaviors that reflect occupational engagement among people with severe mental illness. The construct is sensitive to attentional resources of people with severe mental illness, and thus is sensitive to their prospects of interaction with the immediate environment and occupational engagement. Perhaps the present Rasch analysis in favor of the POES can be explained by the items and scaling, since a detailed text supplements each score, and emanates from empirical time use studies that provide a glimpse of the real life occupations reported by people with severe mental illness.

These results underscore the importance of the POES. The POES describes the extent to which a person can orchestrate a balanced rhythm of activity and rest, a variety and range of meaningful occupations and routines, and has the ability to move within society and interact socially. These findings suggest that occupational engagement occurs, and thus has to be understood, in relation to time. Moreover, occupational engagement involves interpretation and comprehension that emanates from experience, a process that forms the basis for ongoing occupational engagement and a cyclical means of maintaining a sense of self and well-being [1, 9]. Transforming scales, such as the POES, to interval scaling in a valid way is essential to support the evaluation of evidence-based mental health care practice.

Future research should involve psychometric testing in diverse international contexts. Given that the POES is requested by clinicians around the world, from North America, Europe, the Middle East, Asia, and Australia, study of the invariance and construct validity is warranted in other cultural and national contexts. Future research can explore whether the POES is applicable for other primary diagnostic groups, such as people with affective or more common mental disorders. Do such groups have similar or diverse time use patterns? Are they assessed differently with regard to the POES items on occupational engagement? In addition, research on which interventions address and affect changes of the level of occupational engagement in people with severe mental illness would be interesting. We know that vocational rehabilitation with the Individual Placement and Support (IPS) approach has an impact on occupational engagement as assessed by the POES as early as 6 months [13]. At the same time, a 12-week time use and occupation based program, Action Over Inertia, did not reveal such changes in engagement among a smaller group of people with persistent mental illness [14]. Perhaps the challenge of changing the time use patterns and levels of occupational engagement is not an easy task for people with severe mental illness. Rather, a recovery process with time to unfold and establish itself may be needed. Occupational therapy interventions that support changes in time use and occupational engagement, and thus health, need further attention. The POES has a role to fill in such research and practice.

### Limitations

This Rasch analysis was limited to people with severe mental illness living in Sweden. Participants probably reflect a group that is in a stable phase of illness since their overall level of symptoms was low. This is reflected in the POES mean score, which was above the scale midpoint. In addition, the mean score of the BPRS symptom scale was 1.8 (range = 1-3.3). This indicates that although the participants were deemed to have severe mental illness or disabilities, they exhibited few symptoms. It may also reflect the target group of outpatients, where the most common scenario is likely that clients are in psychiatric and medical treatment and/or rehabilitation; in a state where they were willing to participate in a cross-sectional study. The POES fit to the Rasch model indicates that the ability to engage in occupations differed in a systematic way and this most likely reflects symptom severity, as shown in previous research [1].

## Conclusion

Based on modern psychometric calculations the POES scale shows satisfactory internal construct validity. The transformed POES sum score can be used on an interval scale to measure status and changes in occupational engagement in practice and research for people with mental illnesses across age, diagnosis, sex, and country of origin groups. The Rasch analysis supports the unidimensionality of the scale and fits logically with the underlying construct of the occupational engagement continuum.

## Abbreviations

DIF: Differential Item Functioning
POES: Profiles of Occupational Engagement in people with Severe mental illness
PSI: Person Separation Index
BPRS: Brief Psychiatric and Rating Scale
